# Designing rare disease care pathways in the Republic of Ireland: a co-operative model

**DOI:** 10.1186/s13023-022-02309-6

**Published:** 2022-04-11

**Authors:** A. J. Ward, D. Murphy, R. Marron, V. McGrath, M. Bolz-Johnson, W. Cullen, A. Daly, O. Hardiman, A. Lawlor, S. A. Lynch, M. MacLachlan, J. McBrien, S. Ni Bhriain, J. J. O’Byrne, S. M. O’Connell, J. Turner, E. P. Treacy

**Affiliations:** 1 National Rare Diseases Office, Mater Misericordiae University Hospital, Eccles St, Dublin 7, Ireland; 2 Rare Diseases Ireland, Carmichael House, North Brunswick St, Dublin 7, Ireland; 3 European Organisation for Rare Diseases (EURORDIS), Paris, France; 4 Division of Urban General Practice, School of Medicine, University College, Dublin, Ireland; 5 Academic Unit of Neurology, Trinity Biomedical Sciences Institute, Trinity College, Dublin, Ireland; 6 Department of Neurology, Beaumont Hospital, Beaumont, Dublin, Ireland; 7 22Q11 Ireland, North Brunswick Street, Dublin, Ireland; 8 Clinical Genetics, Children’s Health Ireland (CHI) at Crumlin, Dublin, Ireland; 9 School of Medicine, University College Dublin, Dublin, Ireland; 10 School of Medicine, Trinity College Dublin, Dublin, Ireland; 11 Disability Services, Health Service Executive, Dublin, Ireland; 12 Psychology and Social Inclusion, Assisting Living and Learning Institute, Maynooth University, Maynooth, Ireland; 13 Department of General Paediatrics and Neurodisability, CHI at Temple Street, Dublin, Ireland; 14 Office of the National Lead for Integrated Care, Health Service Executive, Dr. Steeven’s Hospital, Dublin, Ireland; 15 National Centre for Inherited Metabolic Disorders, Mater Misericordiae University Hospital, Dublin, Ireland; 16 Department of Diabetes and Endocrinology, Children’s Health Ireland (CHI) at Crumlin, Dublin, Ireland; 17 Department of Paediatrics, Royal College of Surgeons of Ireland (RCSI), Dublin, Ireland

**Keywords:** Rare diseases, Care pathways, Ireland

## Abstract

**Background:**

Rare diseases (RDs) are often complex, serious, chronic and multi-systemic conditions, associated with physical, sensory and intellectual disability. Patients require follow-up management from multiple medical specialists and health and social care professionals involving a high level of integrated care, service coordination and specified care pathways.

**Methods and objectives:**

This pilot study aimed to explore the best approach for developing national RD care pathways in the Irish healthcare system in the context of a lack of agreed methodology. Irish clinical specialists and patient/lived experience experts were asked to map existing practice against evidence-based clinical practice guidelines (CPGs) and best practice recommendations from the European Reference Networks (ERNs) to develop optimal care pathways. The study focused on the more prevalent, multisystemic rare conditions that require multidisciplinary care, services, supports and therapeutic interventions.

**Results:**

29 rare conditions were selected across 18 ERNs, for care pathway development. Multidisciplinary input from multiple specialisms was relevant for all pathways. A high level of engagement was experienced from clinical leads and patient organisations. CPGs were identified for 26 of the conditions. Nurse specialist, Psychology, Medical Social Work and Database Manager roles were deemed essential for all care pathways. Access to the therapeutic Health Service Professionals: Physiotherapy, Occupational Therapy, and Speech and Language Therapy were seen as key requirements for holistic care. Genetic counselling was highlighted as a core discipline in 27 pathways demonstrating the importance of access to Clinical Genetics services for many people with RDs.

**Conclusions:**

This study proposes a methodology for Irish RD care pathway development, in collaboration with patient/service user advocates. Common RD patient needs and health care professional interventions across all pathways were identified. Key RD stakeholders have endorsed this national care pathway initiative. Future research focused on the implementation of such care pathways is a priority.

## Background

In Europe, a rare disease (RD) is defined as a condition with less than five affected persons per 10,000 [1]. It is estimated that 300,000 people in Ireland are living with one of over 6000 known RDs [2]. These conditions tend to be complex, serious, debilitating, chronic and multi-systemic, often associated with physical, sensory and intellectual disability. In many cases patients require follow-up care with management from multiple medical specialists and health and social care professionals, which necessitates a high level of integrated care and service coordination between hospital, community, social and primary care services [3].

People with RDs experience significant challenges in securing diagnoses and accessing appropriate coordinated services, care and treatment [4, 5]. Patients/service users and carers/supporters report that lack of care coordination is a central barrier to accessing timely interventions and has a major impact on health and well-being [6, 7].

Ineffective use of resources due to fragmented services can prolong the ‘diagnostic odyssey’ for many people with RDs [8]. Health care professionals (HCPs) from primary through to tertiary care report a lack of access to RD educational opportunities and struggle to meet the needs of people with RDs [9–11]. Care pathways and defined standards for rare conditions are lacking [12]. Consequently, people with RDs access a disproportionate level of health care resources [13, 14]. The COVID-19 pandemic has further highlighted existing healthcare challenges for people with RDs and the need for more resilient healthcare systems which incorporate telemedicine [15, 16].

Development and implementation of RD care pathways strongly aligns with Irish national health service priorities as outlined in the Model of Care for Rare Diseases 2019 [17], with a focus on delivering more integrated care locally through the national Sláintecare Health Reform Programme 2021–2023 [18].

At a European Union level, member states are committed to facilitating equitable access to timely assessment and diagnosis and high quality, cost-effective care for all people with RDs, and to incorporate rare diseases into social services and policies [19, 20]. In 2017, 24 European Reference Networks (ERNs), virtual networks of healthcare providers across the European Union, were launched to improve RD patient outcomes by enhancing access to specialised education of healthcare professionals (HCPs), developing and endorsing best practice guidelines, providing virtual expert consultations for complex cases and promoting clinical research activity [21]. The ERN Board of Member States (2019) considers the development of national RD patient care pathways as central in the integration of ERNs into member states [22].

Care Pathways are complex interventions that organize care for a well-defined group of patients for a well-defined period [23]. They can be at a system, service or individual care level, have varying levels of granularity and can be multi-professional typically incorporating multiple guidelines [24].

### Methodology for developing a care pathway

Whilst there is internationally agreed best practice methodology for the development of CPGs, there is no commonly recognised methodology for care pathways. Achieving international consensus on care pathway design methodology is complicated by the heterogeneity of national healthcare systems. However, it is agreed that co-design with patient partners is essential [25, 26]. ‘RarERN Path’ has defined a model for care pathway development that is based on capturing existing practice through a narrative medicine approach and achieving patient, carer and HCP consensus on an optimized pathway [27]. Patients accessing evidence-based care have increased health outcomes and their care is more cost effective [28, 29]. Care pathways increase evidence-based care and improve care process coordination [23, 30]. However, identifying robust CPGs for RDs can be challenging due to limited randomized control trial data [31].

This pilot study explored the optimal approach to developing Irish RD care pathways by focusing on 29 multi-systemic conditions. A secondary objective was to determine the resources needed to implement optimum care pathways at the national level although it was evident during the development of the project that the resourcing for this standard of care was not in place at many of the Centres of Expertise.

## Methods

**Fig. 1:**
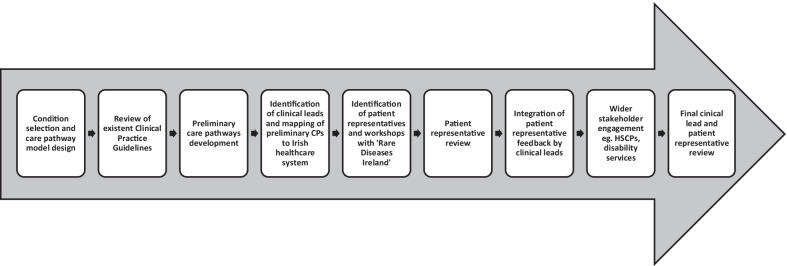
Flow diagram illustrating the development process of the care pathways

**Fig. 2:**
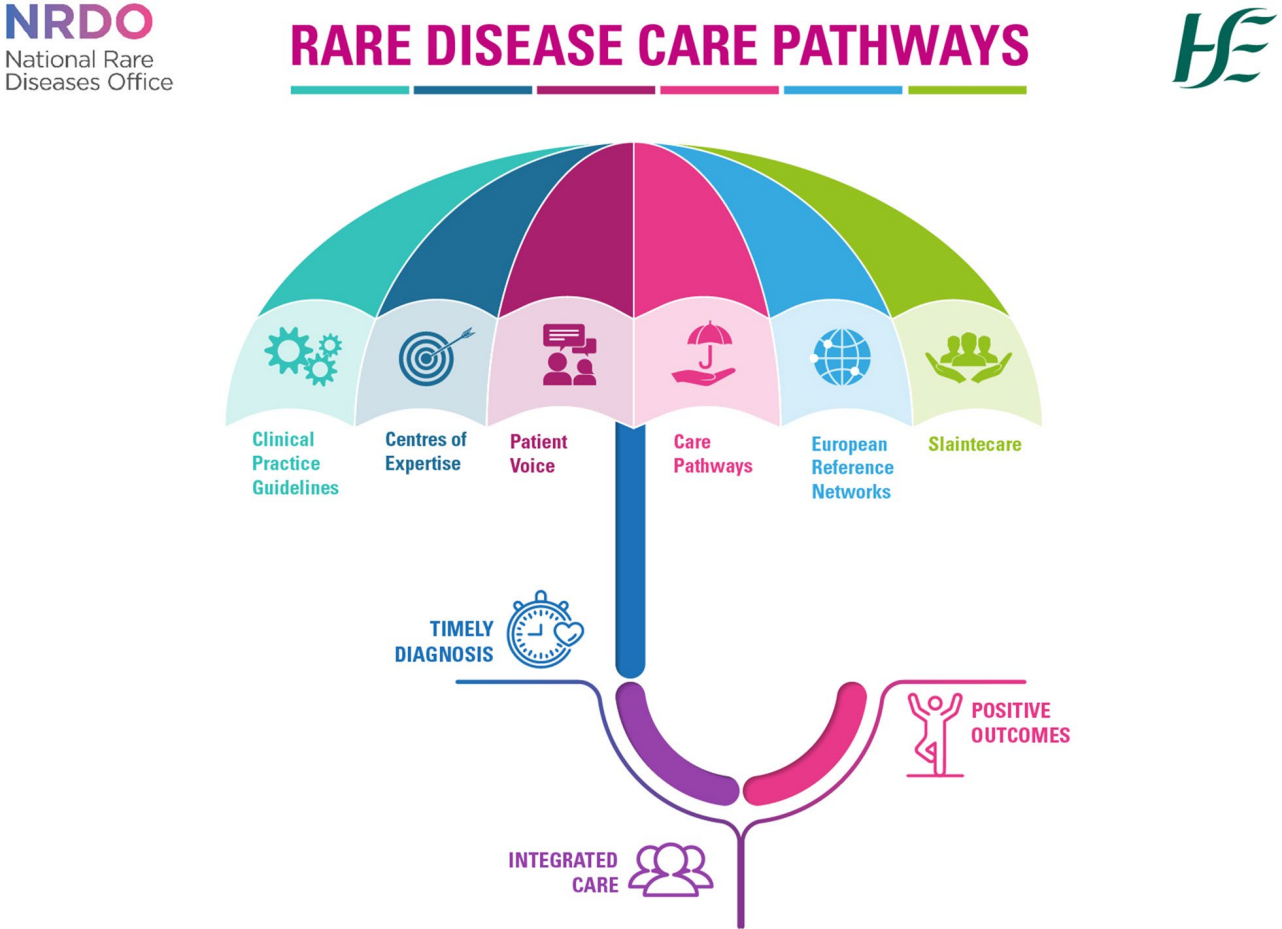
National Rare Disease Care Pathway Collaborative Elements Infographic

**Fig. 3:**
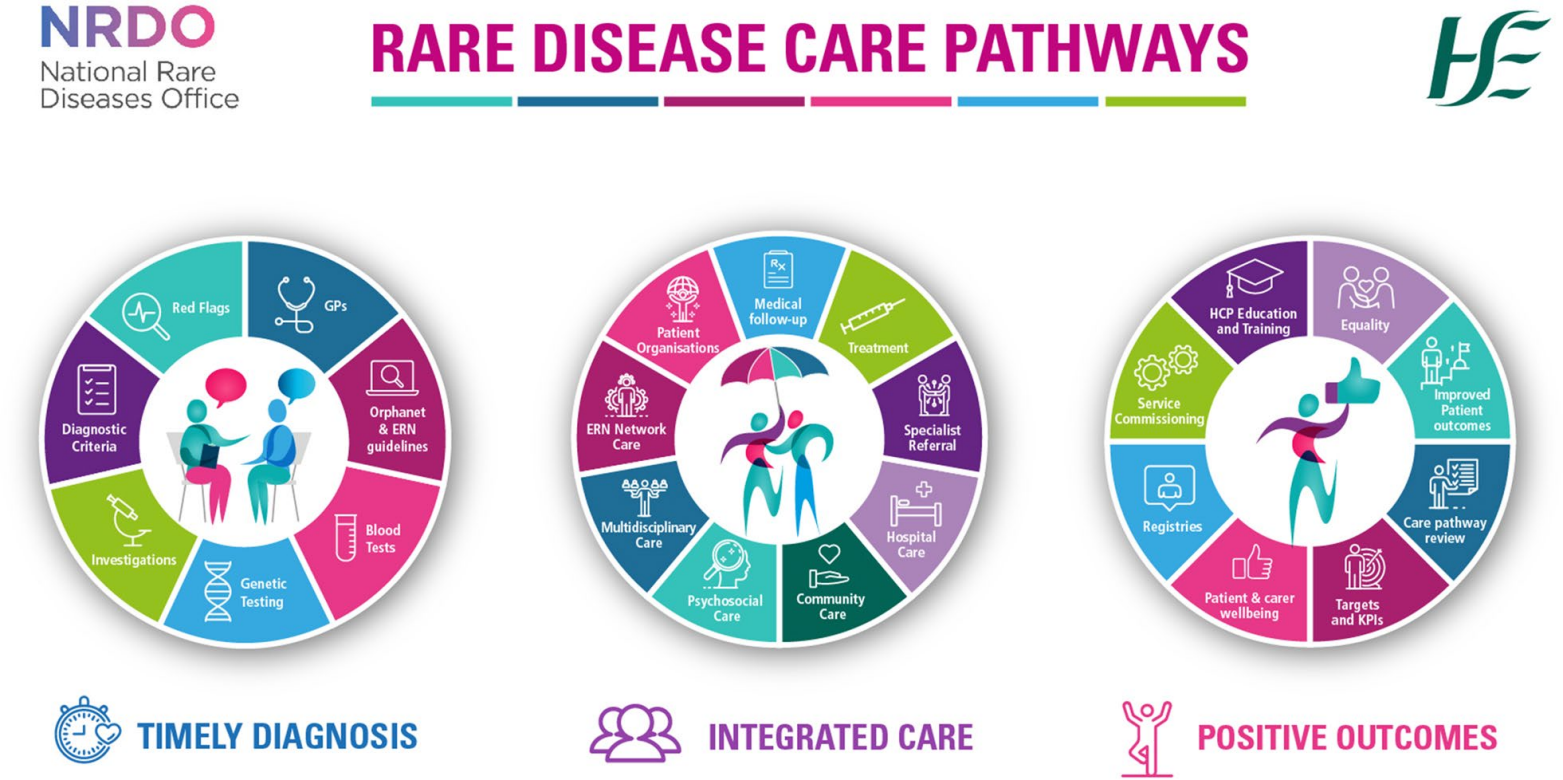
Rare Disease Care Pathway Patient Journey Infographic

Figure 1 summarises the methodology used in the care pathway development process. Figures 2 and 3 summarise the collaborative elements of the pathways and the aims across the patient journey.

### Condition selection

29 rare conditions, across 18 ERNs, were selected due to their estimated higher prevalence in the Irish population, based on preliminary data derived from the Irish ERN applications and Orphanet data [32]. Conditions were considered only if they were multi-systemic and chronic in nature requiring multidisciplinary care and services. Rare cancers and rare infectious diseases were not included, as these do not fall under the remit of the Irish National Rare Diseases Office (NRDO) but are covered, in Ireland, by separate national health service policy, commissioning and clinical governance structures.

### Care pathway model

The initial care pathway model structure was designed based on input from several hospital consultant medical lead collaborators, with four over-arching key categories under which the medical and HSCP disciplines and interventions could be placed and detailed:

- Diagnosis
- Hospital-based specialist care
- Hospital and community-based care
- Primary and community-driven care

**Fig. 4:**
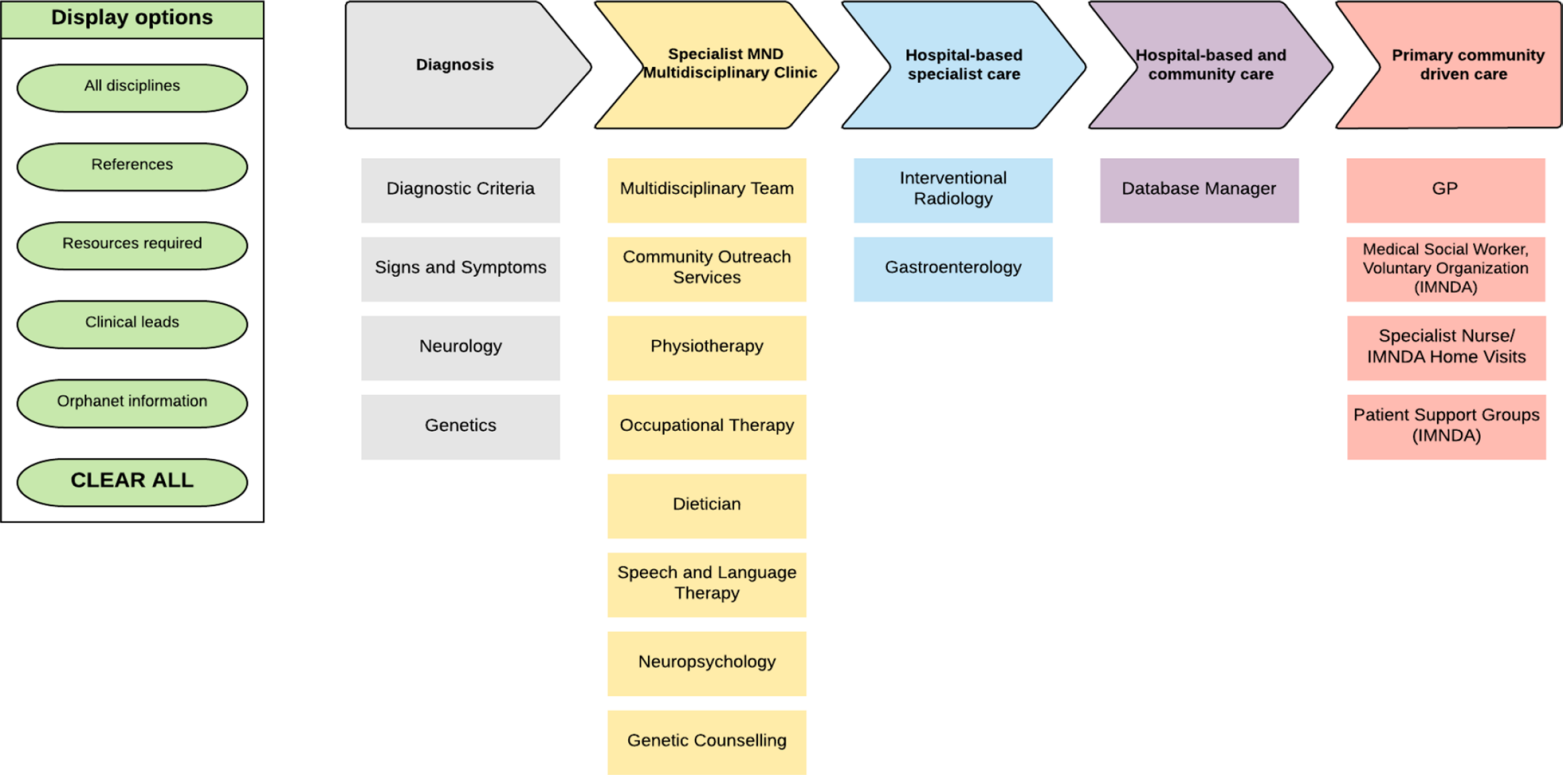
Lucid Chart care pathway model of the Amyotrophic Lateral Sclerosis Care Pathway ref [49] illustrating the required medical disciplines, HSCPs, steps of diagnosis and care. The display options section shows the core information—references, clinical leads, centres of expertise, Orphanet information and resources

For certain conditions, it was necessary to include an additional category to reflect service and support intervention delivery models. For example, a ‘Children’s Disability Network Team (CDNT)’ category was added to the 22q11 deletion syndrome and Angelman syndrome care pathways to capture recently completed reconfiguration within children’s disability services in Ireland. The Amyotrophic Lateral Sclerosis (ALS) care pathway included a specific category called ‘Specialist MND Multidisciplinary Clinic’ to accurately reflect how services for ALS are currently delivered (Fig. 4). To allow the interactive capacity of the model to be realised, a purpose-designed Microsoft ‘Teams’ LucidChart was developed. This allows the details for each section to be revealed and cleared as users move through the pathway by clicking on the drop-down menus to select specific information.

### Preliminary care pathway development

A review of existent CPGs, published within the last 10 years, was performed for each condition by searches of Pubmed, Cochrane, Orphanet and relevant ERN websites. Preliminary care pathways were designed based on the guidelines identified in this evidence-based review, where specific guidelines existed. Detailed information about diagnosis, laboratory testing, investigations, management and treatment, including the frequency of testing or monitoring was recorded under the relevant medical and HSCP disciplines to create a preliminary care pathway for each condition.

### Clinical leads

Expert Irish clinical leads were identified for each condition by liaising with National Reference Network Centres connected under the ERNs as registered on the Irish Orphanet site. For those conditions with possible paediatric onset associated with lifelong care, both paediatric and adult clinical leads were identified. Clinical Leads were asked to map the preliminary care pathways onto existent Irish healthcare services and structures to develop optimal care pathways. The Clinical leads engaged with multidisciplinary teams (MDTs) and professionals from other disciplines for detailing of specific sections, where appropriate.

### Patient representatives

A co-ordinating patient/service user representative was identified for each condition by the Irish national patient rare disease alliance—‘Rare Diseases Ireland’ (RDI) in liaison with the national patient organisations listed on Orphanet. The patient/service user representative co-ordinators for each condition were sent the relevant care pathway document and asked to invite key patient advocates from their network to attend an online workshop jointly led by RDI and NRDO. Co-created support materials were provided. This included an information leaflet and slide presentation providing an overview of the project, guidance on the contribution requested from patient representatives, and a feedback questionnaire. The main objectives of these workshops were: to create a supportive space for collaboration and sharing of experiential knowledge; to better understand the common needs and interventions which patients with rare diseases prioritise; and to enhance the relevance and utility of the care pathways. Additional patient representative support was provided by RDI and NRDO via email, telephone and follow-up video call.

A range of forums for contributions were utilised due to the diverse operational structures of the patient organisations involved: patient representative co-ordinators out-reach within their networks; establishment of short-term working groups; review by patient organisation boards; direct liaison of patient representatives with clinical leads. Patient representative feedback was reviewed by the relevant clinical lead(s) before integration to ensure safe clinical governance over the content. These care pathways were presented to patient representatives for final review.

### Primary care

Engagement with primary care HCPs was sought to ensure that rare disease care pathways best represent the role of General Practitioners (GPs). This aimed to better understand and address the needs of GPs at key points of contact with people with RDs around diagnosis, care and management.

In total, the initial design and guideline review process of the project tool took approximately six months. The next steps of engaging clinical leads and patient representatives has taken approximately eighteen months and is still on-going.

## Results

### Care pathways developed

29 co-produced optimal national rare disease care pathways were developed. Common components and themes across the different pathways were identified to produce a RD care pathway model template. Table 1 illustrates the conditions studied.

**Table 1:** 29 rare conditions selected for care pathway development across 18 ERNs

ERN	Orphanet Disease	ORPHAcode
Endo-ERN	Turner syndrome	99413
Endo-ERN	Primary Adrenal Insufficiency	101958
ERKNet	Glomerular disease	183586
ERN BOND	Osteogenesis Imperfecta	666
ERN BOND	Hypophosphataemic Rickets	437
ERN EpiCARE	Tuberous Sclerosis	805
EuroBloodNet	Sickle Cell Anaemia	232
EuroBloodNet	Von Willebrand disease	903
ERN EURO-NMD	Duchenne Muscular Dystrophy	98896
ERN EURO-NMD	Amyotrophic Lateral Sclerosis	803
ERN EYE	Retinitis Pigmentosa	791
ERN EYE	Usher Syndrome	886
ERN GENTURIS	Neurofibromatosis type 1	636
ERN GUARD-HEART	Long QT syndrome	768
ERN ITHACA	22q11.2 deletion syndrome	567
ERN ITHACA	Angelman syndrome	72
ERN LUNG	Sarcoidosis	797
ERN RARE-LIVER	Wilson Disease	905
ERN ReCONNET	Ehlers-Danlos Syndrome	98249
ERN RITA	Vasculitis	52759
ERN RITA	Juvenile Idiopathic Arthritis	92
ERN-RND	Hereditary Spastic Paraplegia	685
ERN-RND	Early-onset generalized limb-onset dystonia	256
ERN Skin	Inherited Epidermolysis Bullosa	79361
MetabERN	Phenylketonuria	716
MetabERN	Fabry Disease	324
VASCERN	Hereditary Haemorrhagic Telangiectasia	774
VASCERN	Marfan syndrome	558
VASCERN	Vascular Ehlers-Danlos Syndrome	286

### Clinical lead identification

For most childhood-onset conditions requiring lifelong care, both paediatric and adult clinical leads were identified. Our findings identified a significant gap in adult service provision for neurodevelopmental conditions such as Neurofibromatosis Type 1. For certain conditions (e.g., Amyotrophic Lateral Sclerosis), an adult-only care pathway was relevant. The pathway for transition from paediatric to adult services was poorly developed for a number of services with reference to the recommended National Rare Diseases ‘Model of Care’.

### Clinical practice guidelines

CPGs were identified for 26 of the 29 conditions. However, many were based on lower levels of evidence, focused on a single body system, represented the position of a specific professional group, were over 10 years old or were not written in English.

### Core components of a care pathway

**Fig. 5:**
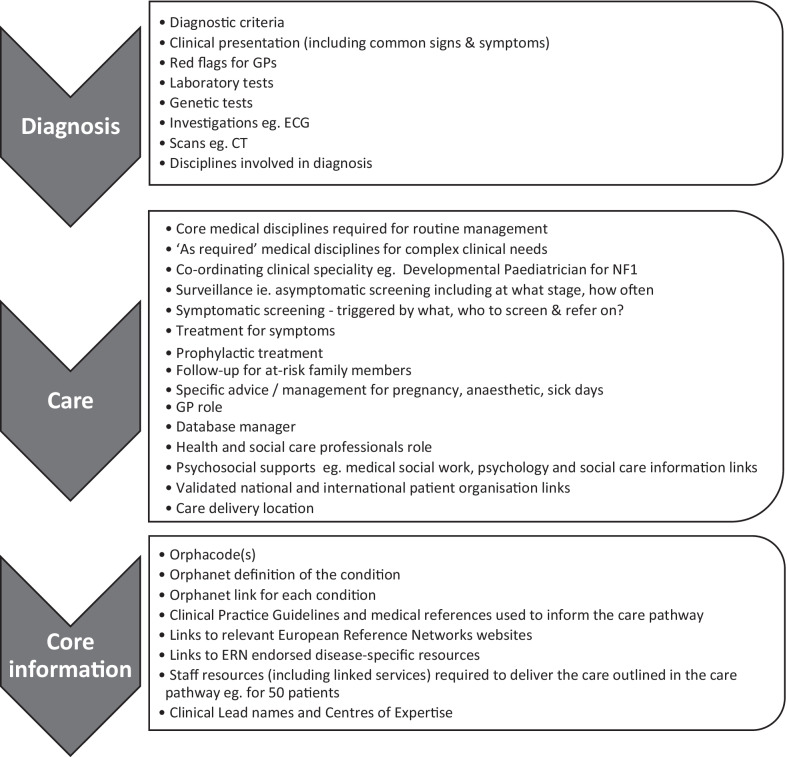
Core components of a care pathway

Whilst the output from Clinical Leads was heterogenous, core components necessary across all care pathways emerged (Fig. 5).

- ‘Diagnosis’ section features included: clinical presenting features; diagnostic criteria; tests and investigations required for diagnosis detailed under the medical disciplines responsible to carry these out; red flags for GPs were included where possible.
- ‘Care’ section features included: medical disciplines and interventions necessary for patient management; symptomatic and asymptomatic screening; triggers for onward referrals; essential HSCP interventions; delineation of the GP role; links to relevant patient organisation(s).
- ‘Information’ section features included: CPGs and references used to inform the care pathway, the Orphanet definition and link for each condition along with Orphacode(s), relevant ERN links and clinical leads information.

‘Resources’ sections were developed containing staff resources and linked services needed to deliver the expected level of care outlined in each pathway, to support their use as a service funding planning tool. This resource section outlined the optimum resources required according for the predicted patient numbers.

### ‘Core’ vs ‘as required’ medical disciplines

Clinical leads indicated the need to distinguish between core medical disciplines and interventions necessary for routine patient management and ‘as required’ (medical disciplines and interventions for patients with more complex clinical needs). For example, core medical disciplines initially identified for Neurofibromatosis Type 1 were Paediatrics, Ophthalmology, Dermatology and Clinical Genetics. However, 11 additional ‘as required’ disciplines including Radiology, Surgery and Oncology were also identified and detailed within the care pathway.

### Key health & social care professional roles

Clinical leads and patient/service user representatives identified a Nurse Specialist, Medical Social Worker, Psychologist and Registry Manager roles as being essential in all 29 pathways (Table 2). Genetic counselling was considered a key role for 93% of pathways as these conditions have a major underlying genetic aetiology. Occupational Therapy, Physiotherapy, and/or Speech and Language Therapy were defined as key in 76% of the care pathways. 45% of pathways included dietetics as a key role.

**Table 2:** Key HSCP roles in holistic care for people with Rare Diseases

Disease name	Nurse Specialist	Physio therapy	Occupational Therapy	Speech and Language Therapy	Psychology	Medical Social Worker	Genetic Counselling	Database Manager	Dietetics
Turner syndrome	✓				✓	✓	✓	✓	✓
Primary Adrenal Insufficiency	✓	✓			✓	✓	✓	✓	✓
Glomerular disease	✓				✓	✓	✓	✓	
Osteogenesis Imperfecta	✓	✓	✓		✓	✓	✓	✓	✓
Hypophosphataemic Rickets	✓	✓	✓		✓	✓	✓	✓	
Tuberous Sclerosis	✓	✓	✓	✓	✓	✓	✓	✓	
Sickle Cell Anaemia	✓	✓			✓	✓	✓	✓	
Von Willebrand disease	✓	✓			✓	✓	✓	✓	
Duchenne Muscular Dystrophy	✓	✓	✓	✓	✓	✓	✓	✓	✓
Amyotrophic Lateral Sclerosis	✓	✓	✓	✓	✓	✓	✓	✓	✓
Retinitis Pigmentosa	✓		✓		✓	✓	✓	✓	
Usher Syndrome	✓	✓	✓	✓	✓	✓	✓	✓	
Neurofibromatosis 1	✓		✓	✓	✓	✓	✓	✓	
Long QT syndrome	✓				✓	✓	✓	✓	
22q11.2 deletion syndrome	✓	✓	✓	✓	✓	✓	✓	✓	✓
Angelman syndrome	✓	✓	✓	✓	✓	✓	✓	✓	✓
Sarcoidosis	✓	✓			✓	✓		✓	
Wilson Disease	✓	✓	✓	✓	✓	✓	✓	✓	✓
Ehlers Danlos—Vascular type	✓	✓	✓		✓	✓	✓	✓	
Ehlers-Danlos Syndrome	✓	✓	✓		✓	✓	✓	✓	
Vasculitis	✓				✓	✓	✓	✓	
Juvenile Idiopathic Arthritis	✓	✓	✓		✓	✓		✓	
Hereditary Spastic Paraplegia	✓	✓	✓	✓	✓	✓	✓	✓	✓
Early-onset generalized limb-onset dystonia	✓	✓	✓	✓	✓	✓	✓	✓	✓
Epidermolysis Bullosa	✓	✓	✓	✓	✓	✓	✓	✓	✓
Phenylketonuria	✓				✓	✓	✓	✓	✓
Fabry Disease	✓				✓	✓	✓	✓	✓
Hereditary Haemorrhagic Telangiectasia	✓				✓	✓	✓	✓	
Marfan syndrome	✓	✓	✓	✓	✓	✓	✓	✓	

### Care coordination

Patient representatives indicated the importance of identifying a HCP for each care pathway to take on a care co-ordinator role. For example, in the case of sarcoidosis 90% of patients present to a respiratory specialist who manages onward referrals to other specialists, as required. In other pathways, the nurse specialist has a central role as the co-ordinator of MDT care, for example in Epidermolysis Bullosa. In the paediatric 22q11 deletion syndrome, a care coordinator is established as a distinct role that can be occupied by a nurse specialist or other relevant HSCP. In the Irish rare disease centres of expertise relevant in this study, the nurse specialist is frequently designated the role of ‘Case Manager’ in liaison with the patient’s respective specialist consultant. In multiple pathways the GP has a central role in care co-ordination particularly in supporting patients to navigate and access local community services for health and social care.

### Patient/service users representatives

Five online workshops involving patient representatives for 14 care pathways were held, co-hosted by Rare Diseases Ireland and the National Rare Diseases Office. Several major themes emerged, including recognition of the challenges and needs common to all rare disease patients, access to co-ordinated care, the priority for holistic care that recognises patients’ and carers’ psychosocial needs, the value of more timely and local community access to HSCPs with a focus on optimising child development and function, the benefit of support for education, employment and social integration, and the need for support to navigate the welfare system. Common themes also emerged from patient representative feedback on individual care pathways: the central role of Nurse Specialists as a primary point of contact, providing education and support for patients and carers and critical liaison with other HCPs; the need for access to psychology support around diagnosis, transition and other major milestones in the patient journey; the key role of HSCPs in fostering wellbeing and quality of life; the value of play therapy to support coping, adaption and compliance; the central role of patient organisations in providing support, information and advice was emphasised across multiple pathways.

## Discussion

A primary objective of this project was to develop an optimal process for the introduction of national RD care pathways into the Irish healthcare system in the absence of a commonly recognised best practice methodology. Consideration of such a broad range of conditions across 18 different ERNs enabled the identification of common components and roles and the development of a national Rare Diseases care pathway model template, which was a fundamental aspect of this study (see Fig. 4, in which the core template was used in relation to Amyotrophic Lateral Sclerosis as an example). The aim was to create an adaptable, interactive, and updatable model with the capacity to outline the coordinated interventions by multiple healthcare providers from pre-diagnosis across the entire life-long patient journey. The model can be flexible to allow for inclusion of specific delivery models where they exist and ensuring adaptability to evolving service configuration. For example, in Ireland, the interdisciplinary Children’s Disability Network Teams have recently been established, comprising over 20 HSCPs, to provide services and supports for children with complex disabilities. Care pathways can be mapped to their respective patient journeys to evaluate if holistic needs of the affected patient population are adequately addressed [33]. Active engagement with health service managers involved in service design, at all stages of care pathway development, facilitates accurate alignment with national service delivery models.

Patient representatives emphasised the importance of a holistic approach to highly specialised care, with a key focus on access to psychosocial care. RD pathways can signpost patients to therapeutic interventions, psychological care and social services, thereby supporting patients and families to navigate education, employment and welfare supports. For example, in the DMD care pathway the medical social worker section details respite care to promote awareness of and access to these support services. This is consistent with findings from published surveys across the wider RD patient community which prioritise improved social inclusion, mental health and quality of life as a means to redress the detrimental impact on personal, professional and socioeconomic status experienced by so many people living with a RD [3, 7, 33]. Patient representatives attributed high value to HSCP and psychosocial roles. As many rare diseases start in childhood and do not yet have effective treatments, the need to optimise child development and function through therapeutic support was considered critical to optimising quality of life. Timely access to local community psychology services was prioritised by patients/service users at key points in the patient journey. Within the Irish healthcare landscape, provision of psychological interventions has tended to be limited to restricted scenarios and often hospital-based; however, psychology services are now provided in each of the 91 Children’s Disability Network Teams that provide community-based services throughout Ireland.

### Gaps in adult services

Challenges in mapping adult Irish clinical experts for lifelong, childhood-onset neurodevelopmental conditions, such as NF1, 22q11 deletion syndrome and Angelman syndrome, revealed significant gaps in adult service provision due to a lack of clear transition pathways. Consequently, GPs are often left to coordinate ongoing management in *an ad* hoc manner [10]. The lack of adult multidisciplinary care to address the complex medical and psychosocial needs of these patients is a primary concern for patients and carers, who often take on the role of care coordinator themselves [6]. A focus on delivering adequate co-ordinated adult care for life-long neurodevelopmental conditions is required [34].

### Primary care

The role of general practice in the diagnosis, treatment and ongoing care of people with RDs and the need for better communication, more consistent coding nomenclature, shared electronic health records and enhanced education in primary care has been highlighted [10]. In Ireland, the establishment of multidisciplinary primary care teams has been core to health policy since 2001 and their role in the care of patients with rare diseases is central [12, 17, 35]. Furthermore, the value of enhanced and seamless communication between specialist centres and primary care HCPs in managing patients with rare diseases is consistent with the integrated approach to healthcare delivery that is a core part of the Irish ‘Slaintecare’ reform programme [18].

National RD care pathways can address the self-reported gaps in primary care RD education by facilitating access to reliable RD resources; delineating the role of GPs in RD diagnosis and management; mapping local healthcare system organization for RDs including national CoEs; integrating ERNs for expert opinion and network care [11, 35]. They enable local care to be informed by the latest evidence base leading to improved patient outcomes and healthcare service efficiency [33, 36]. Further development of care pathways to include red flags for GPs, mapping of diagnostic testing services and local referral routes will ensure these gaps are bridged.

### Patient partnership

Patient involvement in the development of guidelines and pathways has been reported to enhance the relevance, practicality, and impact of care organised under these clinical support tools [25, 37]. Despite the highly heterogeneous nature of rare diseases, the project found through the active engagement of patient representatives, that people living with a rare disease face common needs and challenges. Of importance is the awareness of and access to healthcare professionals and services with sufficient knowledge of their rare condition. This confirms similar findings evidenced across the wider RD community [38]. Whilst both patients and professionals have a shared goal, they can hold different but equally valuable perspectives. Building mutual respect for both perspectives on care decisions and service design is fundamental to aligning healthcare services on the needs of the patient locally [25]. Notable barriers for meaningful patient-professional partnership are two-fold. Firstly, a perceptional barrier by clinicians who question the value of patient collaboration. However, overtime clinicians have been reported to develop a more positive view on patient involvement [39]. Secondly, patients can question how they can best contribute to the development of guidelines and care pathways due to the use of complex medical terminology. These barriers can be overcome but time and tools are needed to support patient-professional partnerships. Patients’ insights are invaluable as they are ‘experts living with the condition’ and can provide a different perspective to that of clinicians [40, 41].

Patient involvement provides the weight of patients’ opinions and preferences of interventions and treatment, informed by the benefits and harms associated with treatments; empowering patients in decision making about their care; ensuring the holistic needs are understood and addressed [40]. Recognition of patients as experts-by-experience with the capacity to co-design and lead in the development of patient-centred care is central and has been evidenced by the European Patient Advocacy Groups (ePAGs) in ERNs.

As Ireland is in the initial stages of ERN membership, ePAG membership is in early phase development. Although Ireland has an active rare disease patient organisation base, the population size of 5 million means that many rare diseases do not have an Irish patient organisation. Our study found that involving ‘Rare Diseases Ireland’ (RDI) as key facilitators was effective in mapping patient representative and supporting their contribution. For several conditions, where no specific Irish patient organisation exists, clinical leads were asked to nominate an appropriate patient representative. ‘RDI’ worked to support these individuals and also liaised with relevant UK support groups to identify possible additional Irish patient representatives, as on occasion Irish patients join UK support groups where no group is available in Ireland.

### Provision of genetic services

Over 70% of RDs have a significant genetic basis [32]. Also, for RDs with a non-genetic basis there can be significant heritability, indicating the likelihood of genetic susceptibility and/or rare genetic sub forms. Genetic counselling was considered a core discipline for most of the pathways. As the clinical genomics landscape evolves due to improving diagnostic methods and treatments, care pathways need to be flexible to adapt [42]. The genetic counselling profession is uniquely placed to support these transitions in best practice and to ensure that ethical principles are followed that consider the implications for patients and their extended family members. As genomics promises to deliver powerful diagnostic solutions, national clinical genetic services struggle to absorb increased demand [43]. Mainstreaming of genetic testing is the inevitable consequence. However, the inherent risks of misinterpretation of complex genomic data are evident even for HCPs with formal clinical genetics training and extensive genomic experience [44]. Clinical Geneticists and Genetic Counsellors have a central role in education of non-Genetics HCPs to promote the safe delivery of genomic medicine [45].

### Registries

Inclusion of database managers for registry development and curation was deemed a core requirement in line with ERN priorities. Registries are recognised as an invaluable resource for capturing epidemiological disease information and natural history, identifying patient cohorts available for clinical research, assessing therapeutic outcomes, generating evidence and monitoring CoEs for disease-specific key performance indicators that can be used to gauge ERN activity and impact. Centralised registry development to ensure data interoperability and uniform database structure is essential for integration into the ERN IT system [46]. The use of Orphacodes within our care pathways promotes aligned codification with ERN RD registries, which are committed to Orphacode designation as a key parameter, and implementation of the EC eHealth Network Guideline on the electronic exchange of health data under the Cross Border Directive 2011/24/EU [47].

### Accessibility and dissemination

The ability to recognise RDs at initial points of contact with patients is a significant challenge [33]. Support is also required for primary and secondary care HCPs to ensure safe patient management while patients wait to access specialist tertiary services. Ready access to care pathways at key points of contact across frontline clinical services is critical to maximise their utility. To facilitate this, it is envisaged in our programme that the care pathways will be hosted on a dedicated website which will be accessible through the National Rare Disease Office, Irish Health Service Executive, Orphanet, relevant ERN and national professional and patient organisation websites. This aligns with physicians’ preferences for professionally endorsed channels for accurate RD information dissemination via expert centres and professional associations [11]. The positive level of patient engagement in this study augurs well for co-promotion and effective dissemination of care pathways within the RD patient community via patient organisation networks.

By providing access to national care pathways via a dedicated website, the interactive capacity of the pathways can be fully realized. Future work will focus on the development of patient-friendly versions and tools to enhance patient access by building on these interactive features. The ‘Rare 2030’ recommendations promote enhanced visibility of best practice guidelines and the importance of accessibility for patients by including a patient ‘lay’ summary that should be co-designed and developed with patient and service users [7].

Centralised, updatable versions of each care pathway will facilitate easy access to the latest guidelines by multiple disciplines across different locations which aligns with the ERN eHealth goal of ensuring the availability of up-to-date information [48]. It is proposed that regular future audit by Clinical Leads and Patient Representatives will ensure that new evidence emerging around diagnostics, management and treatment will be captured.

### Implementation

The impact of developing aspirational care pathways, with a significant number that are not implemented to date, was noted by patient representatives and HCPs as a concern. Clinical leads have assessed and detailed the staff resources and linked services required to deliver the level of care outlined in each pathway. This will enable advocacy for commissioning. Significant barriers to successful implementation within local and national services exist with pressures on healthcare systems and demands on clinical time. This study also shows that care pathway development is a time intensive activity requiring dedicated funding to ensure sustainability.

### Limitations of the study

Our methodology aligns with that of the RarERN Path as a framework for collaboration with national clinical experts and patient representatives [27]. However, cost analysis of current and proposed care pathways was not within the scope of this study. Detailing of each care pathway by each discipline involved in care would be optimum. However, a pragmatic approach was taken in this study by selecting the most relevant professionals in the specific care pathway.

### Next steps

To ensure alignment with national healthcare strategies and effective implementation, further engagement with key national health service stakeholders including primary care, integrated care, digital E-health and disability services is ongoing. Further HSCP professional engagement will focus on the key roles identified across pathways to ensure their accurate representation.

The next steps include piloting of individual pathways with follow-up evaluation and revisions for further optimisation. The evaluation will involve benchmarking against the HSE Model of Care for Rare Diseases, which will no doubt highlight barriers to implementation and gaps in service provision such as the lack of adult services for neurodevelopmental conditions [17]. Development of clear transition pathways for all care pathways where the condition has a possible childhood onset will be a priority. Transition planning is detailed within the Nurse specialist role. For certain conditions such as PKU where the numbers of patients transitioning to adult services is significant, the care pathway specifies that a transition coordinator is a key role.

The improved efficiencies of these pathways through shortening the diagnostic journey, enhancing access to appropriate management and intervention and improved patient outcomes, will be require confirmation.

The goal of the project was development of a RD care pathway model and methodology. This will be used as a template for future care pathway progression for further rare diseases, rare disease groups and ultra-rare conditions, a number of which have commenced.

The development of a generic care pathway for undiagnosed RD patients may be beneficial but was outside the scope of this project. Such a pathway aligns with the current Irish national disability service development ‘needs-based’ strategy, the development of national clinical genetics/genomic services and the ‘European Joint Programme on Rare Diseases’ research initiatives.

### Rare 2030 recommendations

The ‘Rare 2030 Foresight’ study emphasises the full development and implementation of national RD plans as a primary recommendation with the development of care pathways as key component [7]. Specifically, digital care pathways can facilitate the collection and evaluation of patient data, by auditing the outcomes and further developing interventions within each pathway based on outcomes. This equips frontline clinical services to deliver more cost-effective evidence-based medicine and personalised care leading to better patient outcomes. Furthermore, digital care pathways have the potential to accelerate the development and uptake of RD treatment options by facilitating European-wide clinical trials and research. National care pathway development can promote care co-ordination between HCPs, improve access to specialists and enhance treatment opportunities as highlighted as the top three highest unmet needs by the RD community to be addressed by 2030 [7].

## Conclusion

This pilot study explored the development of an optimal process for national RD care pathway design in the absence of agreed, common, best practice methodology. Partnership with RD patient representatives and service users was central to the delivery of this study. The high level of engagement experienced from clinical leads and patient organisations highlights the strong endorsement of this care pathway initiative by key RD stakeholders. RD patient needs and health care professional interventions common across all pathways were identified, highlighting the core roles of therapeutic HSCPs, Nurse Specialist, Psychology, Medical Social Work, Database Manager and Genetic Counselling for delivering optimum rare disease care. Effective care pathways offer the best opportunity to translate best practice, evidence-based guidelines into local healthcare services leading to service redesign and more targeted resources to optimise patient health outcomes. It is intended that care pathways will prove to be effective educational, clinical and advocacy tools for general practitioners (GPs), hospital specialists, HSCPs, health service managers, patients and carers. Future research examining the implementation of such care pathways is a priority.

## Data Availability

Not applicable.
